# Metabolomics-transcriptomics joint analysis: unveiling the dysregulated cell death network and developing a diagnostic model for high-grade neuroblastoma

**DOI:** 10.3389/fimmu.2023.1345734

**Published:** 2024-01-04

**Authors:** Wancun Zhang, Mengxin Zhang, Meng Sun, Minghui Hu, Muchun Yu, Jushan Sun, Xianwei Zhang, Bang Du

**Affiliations:** ^1^ Health Commission of Henan Province Key Laboratory for Precision Diagnosis and Treatment of Pediatric Tumor, Children’s Hospital Affiliated to Zhengzhou University, Zhengzhou, China; ^2^ Henan International Joint Laboratory for Prevention and Treatment of Pediatric Disease, Children’s Hospital Affiliated to Zhengzhou University, Zhengzhou, China; ^3^ Henan Key Laboratory of Children’s Genetics and Metabolic Diseases, Children’s Hospital Affiliated to Zhengzhou University, Zhengzhou, China

**Keywords:** neuroblastoma, metabolomics, transcriptomics; therapeutic target, network, diagnostic model

## Abstract

High-grade neuroblastoma (HG-NB) exhibits a significantly diminished survival rate in comparison to low-grade neuroblastoma (LG-NB), primarily attributed to the mechanism of HG-NB is unclear and the lacking effective therapeutic targets and diagnostic model. Therefore, the current investigation aims to study the dysregulated network between HG-NB and LG-NB based on transcriptomics and metabolomics joint analysis. Meanwhile, a risk diagnostic model to distinguish HG-NB and LG-NB was also developed. Metabolomics analysis was conducted using plasma samples obtained from 48 HG-NB patients and 36 LG-NB patients. A total of 39 metabolites exhibited alterations, with 20 showing an increase and 19 displaying a decrease in HG-NB. Additionally, transcriptomics analysis was performed on NB tissue samples collected from 31 HG-NB patients and 20 LG-NB patients. Results showed that a significant alteration was observed in a total of 1,199 mRNAs in HG-NB, among which 893 were upregulated while the remaining 306 were downregulated. In particular, the joint analysis of both omics data revealed three aberrant pathways, namely the cAMP signaling pathway, PI3K-Akt signaling pathway, and TNF signaling pathway, which were found to be associated with cell death. Notably, a diagnostic model for HG-NB risk classification was developed based on the genes *MGST1*, *SERPINE1*, and *ERBB3* with an area under the receiver operating characteristic curve of 0.915. In the validation set, the sensitivity and specificity were determined to be 75.0% and 80.0%, respectively.

## Introduction

Neuroblastoma (NB), originating from the embryonic neural crest, represents the most prevalent extracranial malignant tumor in pediatric patients. It is characterized by an insidious onset and rapid progression, contributing to 8% of childhood cancer-related morbidity and 15% of childhood cancer-related mortality (1, 2). According to the International Neuroblastoma Staging System (INSS), NB can be classified into stages 1, 2A, 2B, 3, 4, and 4S based on an analysis of the primary organ and metastatic sites. It has been observed that patients younger than 1 year of age exhibit a significantly higher 4-year overall survival rate (98.5%) for INSS stage 1, 2A, 2B, 3 diseases compared to patients with stage 4 disease (73.1%). Furthermore, in patients older than 1 year, the NB survival rates at the end of four years were found to be perfect (100%) for stages 1, 2A, 2B, and 3, while it was recorded as only around half (48.5%) for those in stage 4 (3). These distinct stages exhibit significant variations in terms of mortality rates and prognostic outcomes. Therefore, investigating the disparities between high-grade neuroblastoma (HG-NB) (stage 4) and low-grade neuroblastoma (LG-NB) (stages 1, 2, 3) will not only enhance comprehension of the biological functionality of HG-NB but also contribute to refining therapeutic strategies for aggressive NB.

The absence of an efficacious therapeutic targets and diagnostic model for HG-NB constitutes the primary determinant underlying its significantly inferior survival rate compared to LG-NB (4). Hu et al. utilized gene chip and reverse transcription-polymerase chain reaction (RT-PCR) technology to analyze a cohort of clinically diagnosed pulmonary tuberculosis patients, microbiologically confirmed pulmonary tuberculosis patients, non-tuberculosis controls, and healthy controls. They identified candidate lncRNAs with differential expression and established an early diagnosis model to facilitate the early identification of pulmonary tuberculosis (5). Furthermore, in order to identify novel biomarkers suitable for the diagnosis and treatment of prostate cancer, Maik et al. conducted a comprehensive genome-wide transcriptome sequencing analysis on tissue samples obtained from 40 patients with prostate cancer and 8 individuals with benign prostatic hyperplasia. Their findings revealed that TAPIR-1 and -2 play a pivotal role in the pathogenesis of prostate cancer, thereby offering valuable insights for accurate diagnosis and targeted therapeutic interventions (6). Therefore, the systematic investigation of HG-NB to identify its diagnostic biomarkers and therapeutic targets is anticipated to enhance the survival rate of patients with HG-NB.

The emergence of omics has significantly contributed to the advancement of disease diagnosis and treatment, which is highly noteworthy. The field of metabolomics aims to comprehensively characterize the entirety of small molecules present in a given sample, with the ultimate goal of accurately reflecting the intricate metabolic characteristics associated with disease states. This approach holds immense potential for unraveling the underlying pathophysiological processes driving disease progression and facilitating the discovery of novel biomarkers crucial for disease diagnosis and prognosis (7). Dong et al. conducted an investigation into the correlation between pre-diagnostic plasma metabolomics, and the risk of colorectal cancer precursors. Their findings suggest that lipid metabolism and the microbial metabolite phenylacetylglutamine may play a potential role in the early stages of colorectal cancer development (8). Furthermore, Xu et al. conducted targeted metabolomics analysis on a cohort of 86 patients with benign breast lesions and 143 patients diagnosed with breast cancer, aiming to investigate the plasma characteristics associated with breast cancer. A total of 716 metabolites were identified, revealing serotonergic synapses as the predominant differential metabolic pathway (9). Transcriptomics employs high-throughput sequencing techniques to investigate the complete set of transcribed mRNAs within specific cells, tissues, or individuals at a given time and state. This comprehensive approach enables the identification of disparities in gene expression and structure across distinct functional states, thereby elucidating underlying molecular mechanisms (10, 11). Qi et al. employed transcriptome sequencing technology to analyze 5 pairs of endometrial cancer tissues and normal endometrial tissues, revealing downregulation of *ID1*, *IGF1*, *GDF7*, *SMAD9*, *TGF-β*, and *WNT4* expression alongside upregulation of *GDF5*, *INHBA*, and *ERBB4* in endometrial cancer. Furthermore, alterations were observed in the *TGF-β* signaling pathway as well as the PI3K-Akt and estrogen pathways among others. These findings contribute to a deeper understanding of the underlying mechanisms driving endometrial cancer (12). Additionally, transcriptome analysis conducted by Ren et al. revealed that *GPNMB* serves as a promising target in gastric cancer and exerts a crucial positive regulatory role in tumor progression. Moreover, *GPNMB* exhibits diverse regulatory effects on gastric cancer-mediated immunosuppression (13). Furthermore, the integration of transcriptomics and metabolomics has emerged as a robust methodology that enhances comprehension of the potential biological functions and molecular mechanisms underlying diseases (14). In particular, Ren et al. discovered metabolic pathway alterations in prostate cancer by combining metabolomics and transcriptomics, and found abnormal expression of cysteine ​​and methionine metabolism, nicotinamide adenine dinucleotide metabolism and hexosamine biosynthesis. In addition, the metabolite sphingosine exhibited high specificity and sensitivity in distinguishing prostate cancer from benign prostatic hyperplasia, promoting the development of new diagnostic biomarkers and therapeutic targets, which will help to distinguish prostate cancer from benign prostatic hyperplasia (15). Additionally, Zhao et al. investigated the anti-tumor mechanism of tadalafil in human colorectal cancer cells through an integrated analysis of metabolomics and transcriptomics, revealing that perturbations in alanine, aspartic acid, and glutamate metabolism may underlie the primary mode of action for tadalafil’s anti-tumor effect (16). Therefore, the integration of metabolomics and transcriptomics holds significant potential for application in HG-NB, enabling the identification of altered metabolic pathways and diagnostic biomarkers, facilitating the establishment of early diagnosis models, and identifying novel therapeutic targets for HG-NB.

In this study, we conducted a metabolomics analysis of a total of 84 plasma clinical samples and 51 clinical NB tissue samples, integrating metabolomics data with transcriptomics data to perform a comprehensive network analysis of NB. Our aim was to explore the aberrant pathways associated with HG-NB and develop a diagnostic model based on potential biomarkers. The innovation of this study can be summarized as follows (1): Through a systematic analysis, we evaluated the transcriptomics and metabolomics differences between LG-NB and HG-NB to unveil the dysregulated network specific to HG-NB. (2) A noninvasive plasma-based diagnostic model of HG-NB was established. The novel discovery of the dysregulation network associated with HG-NB and the development of the NB diagnostic model are expected to have significant implications for early diagnosis of HG-NB and the future advancement of targeted therapies.

## Materials and methods

### Moral approval

After collection and processing from October 2018 to January 2022, a total of 84 plasma samples (48 cases of HG-NB and 36 cases of LG-NB) and 51 NB tissue samples (31 cases of HG-NB, 36 cases of LG-NB) were obtained from Henan Children’s Hospital. The inclusion criteria included: (1) confirmed pathological diagnosis of NB; (2) clinical assessment of risk grade based on the INSS classification; (3) obtaining informed consent from children or their parents. The exclusion criteria included the following: (1) presence of complications related to other diseases; (2) absence of signed informed consent from either the children or their parents. Plasma samples were collected from NB patients’ fasting plasma in the morning of surgery and promptly frozen at -80°C for subsequent metabolomics analysis. Tissue samples from NB tumors were obtained during surgical resection and immediately stored in liquid nitrogen for transcriptomics analysis. The results presented in **Supplementary Tables 1**, **2** indicate that there were no statistically significant differences observed in terms of age, gender and *MYCN* amplification between HG-NB and LG-NB. However, a notable distinction was found regarding the gross tumor volume and radiological risk factors among the HG-NB and LG-NB samples. This study was reviewed and approved by the committees of Henan Children’s Hospital.

### Metabolomics analysis via high performance liquid chromatography-mass spectrometry

The plasma samples were retrieved from storage at -80°C and promptly thawed in a refrigerator set at 4°C. Following 10 seconds of vortexing, 150 μL of plasma was transferred to a microcentrifuge tube with a capacity of 1.5 mL, followed by the addition of 450 μL acetonitrile maintained at 4°C. After vigorous vortexing for 5 minutes at a speed of 3000 r/min, the mixture was subjected to centrifugation at 13000 r/min for 15 minutes (at a temperature of 4°C). Subsequently, careful extraction yielded a supernatant volume of approximately 300 μL. The stability of the overall experimental results was assessed by preparing quality control (QC) samples, which were obtained by combining equal amounts of supernatant from all samples. An Agilent 6210 time-of-flight MS system equipped with an Agilent 1100 HPLC, a photodiode array detector, and a high-resolution-time-of-flight-MS with an electrospray ionization source was used for the analysis of all extracts. Chromatographic separation was carried out on an Agilent Poroshell 120 EC - C18 (2.7 μm, 3.0 × 100 mm) column. The metabolomics data were collected using the following conditions: mobile phase consisting of A = 0.1% formic acid in water and B = 0.1% formic acid in acetonitrile, with elution conditions as follows: 0 - 3 min, gradient from 5% to 60% B; 3 - 25 min, gradient from 60% to 90% B; 25 - 30 min, gradient from 90% to 100% B; and finally, a constant flow of pure solvent B for the remaining time (30 - 40 min). Experimental settings included an injection volume of 10 μL, column temperature maintained at a constant value of 30°C, and a flow rate set at a steady rate of 0.3 mL/min. MS was performed under both negative and positive ionization modes using nitrogen as drying gas at a temperature of approximately 325°C with a flow rate set at 12 L/min and atomization pressure maintained at 35 psi. Capillary voltage was adjusted to 4,000 V for positive mode and 3,500 V for negative mode while fragmentation voltage was set to 215 V for positive mode and 175 V for negative mode with separator voltage fixed at 60 V. The mass acquisition range encompassed all negative ions within the range of 0.05 - 1.5 KDa.

The samples were subjected to HPLC-MS analysis in order to obtain the raw data files. Agilent Masshunter HPLC-MS software was utilized for converting the original data files into a standardized format. XCMS software package, implemented on the R language platform, was employed for retention time (RT) calibration, peak identification, noise filtration and peak matching of the acquired.mzData format files. Additionally, it allowed setting permissible deviations for both mass-to-charge ratio and RT (mass/charge ratio tolerance = 0.025DA, RT tolerance = 0.5 min). The metabolites exhibiting a RT deviation of 0.5 min and a mass number deviation of 0.025 Da were considered to be identical metabolites. Subsequently, a data matrix comprising mass/charge ratio, RT, peak area, and other relevant information was obtained. Metabolite identification involved the utilization of both primary and secondary MS techniques. Initially, the acquired primary MS information underwent targeted secondary MS analysis to acquire supplementary MS information that served as a reference for subsequent qualitative analysis. Furthermore, by leveraging the precise mass numbers of excimer ions such as [M^+^H]^+^ ions and high-resolution target MS/MS spectra in conjunction with fragmentation patterns observed across various metabolites, potential structures for differential metabolites were deduced through comprehensive analyses involving online databases (METLIN: http://metlin.scripps.edu/, HMDB: http://hmdb.ca/) as well as literature retrieval methods.

The metabolomics analysis was performed using MetaboAnalyst (https://www.metaboanalyst.ca/MetaboAnalyst/home.xhtml), which included partial least-squares discrimination analysis (PLS-DA), heatmap, volcano map, enrichment analysis, pathway analysis, and identification of biomarkers.

### Transcriptomics profiling using RNA-sequencing analysis

The total RNA was extracted using TRIzol reagent following the manufacturer’s protocol. RNA purity and quantification were assessed using the NanoDrop 2000 spectrophotometer (Thermo Scientific, USA). RNA integrity was evaluated using the Agilent 2100 Bioanalyzer (Agilent Technologies, Santa Clara, CA, USA). The libraries were prepared utilizing the TruSeq Stranded mRNA LT Sample Prep Kit (Illumina, San Diego, CA, USA) according to the manufacturer’s instructions. Transcriptome sequencing and analysis were performed by OE Biotech Co., Ltd. (Shanghai, China).

The libraries were sequenced using an Illumina HiSeq X Ten platform, generating 150 bp paired-end reads. Each sample yielded approximately 48.349 million raw reads. The raw data (in fastq format) underwent initial processing with Trimmomatic 18 to remove low-quality reads, resulting in the acquisition of clean reads. Approximately 47.459 million clean reads per sample were retained for subsequent analyses. These clean reads were then aligned to the human genome (GRCh38) using HISAT2 (17).

Fragments per kilobase of exon model per million mapped fragments (FPKM) (18) of each gene was calculated using Cufflinks (19) and the read counts of each gene were obtained by HTSeq-count (20). Differential expression analysis was performed using the DESeq (2012) R package (21). The threshold for significant differential expression was set at *P* value < 0.05 and | log_2_ (fold change) | > 1. Hierarchical cluster analysis was performed to illustrate the gene expression patterns across different groups and samples. Open-access databases, such as Gene Ontology (GO), Kyoto Encyclopedia of Genes and Genomes (KEGG), MetaboAnalyst, Human Metabolome Database, and National Center for Biotechnology Information were utilized to identify metabolic pathways.

### Joint analysis of the metabolomics and transcriptomics

Finally, comprehensive transcriptomics and metabolomics analyses were conducted using MetaboAnalyst 5.0 to perform topological analysis through the joint-pathway analysis module. Official gene symbols and compound names, along with optional fold changes, were entered to evaluate the potential significance of individual molecules (i.e., nodes) based on their network position. Topological analysis assesses the potential significance of a specific molecule (node) based on its position in the pathway and determines its impact value. Degree centrality quantifies the number of connections that are linked to a specific node, while betweenness centrality measures the quantity of shortest paths from all nodes to others that pass through a given node. Closeness centrality gauges the overall distance between a given node and all other nodes. The hypergeometric test was selected for enrichment analysis, degree centrality was chosen as the measure of topology, and combined queries were employed as an integration method.

### Revealing plasma potential biomarkers between HG-NB and LG-NB

The 6 differential genes were selected as potential candidate biomarkers based on transcriptomics results and literature review. Primers for the identified differential genes were designed using the National Center for Biotechnology Information (www.ncbi.nlm.nih.gov) website (**Supplementary Table S6**). RT-PCR was conducted following the instructions provided with HiScript III All-in-one RT SuperMix kit and AceQ qPCR SYBR Green Master Mix kit (Vazyme, Nanjing). The housekeeping gene *NAGK* was chosen as an internal control to normalize mRNA abundance levels. Fold changes in target gene mRNA expression were calculated using the formula 2^−ΔΔCt^.

### Develop the risk diagnostic model of NB

The logistic regression analysis was employed to establish the regression equation for the test set, which consisted of 21 cases of HG-NB and 20 cases of LG-NB. Subsequently, validation against the validation set, comprising 20 cases of HG-NB and 10 cases of LG-NB, was conducted. The data were processed using SPSS 25.0, while Origin 2021 was employed for mapping purposes.

## Results

### The research procedure

**Scheme 1:**
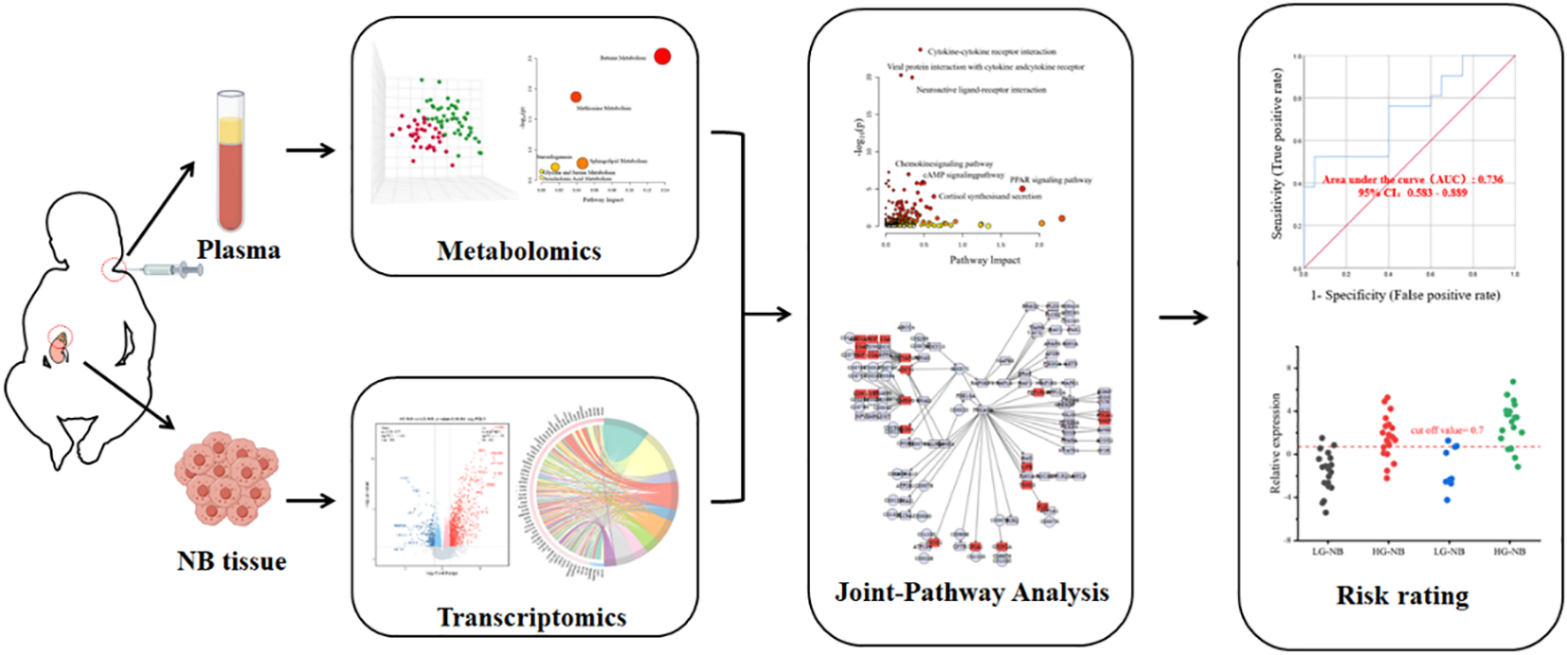
Outline of research method.

The general concept of this study is illustrated in **Scheme 1**. Metabolomics analysis was conducted on a total of 84 plasma samples, comprising 48 cases of HG-NB and 36 cases of LG-NB. Additionally, transcriptomics analysis was performed on 51 NB tissue samples, including 31 cases of HG-NB and 20 cases of LG-NB. Through the integration of metabolomics and transcriptomics data, employing PLS-DA, heatmap visualization, enrichment analysis, pathway analysis, and other analytical approaches, we comprehensively investigated the aberrant pathway network associated with HG-NB and identified potential clinical therapeutic targets. Meanwhile, a risk diagnostic model was established to facilitate early detection of HG-NB.

### The Metabolome differences between HG-NB and LG-NB

To investigate the disparities in metabolites between HG-NB and LG-NB, an initial plasma metabolomics analysis was performed utilizing a non-targeted approach based on metabolomics.

**Figure 1:**
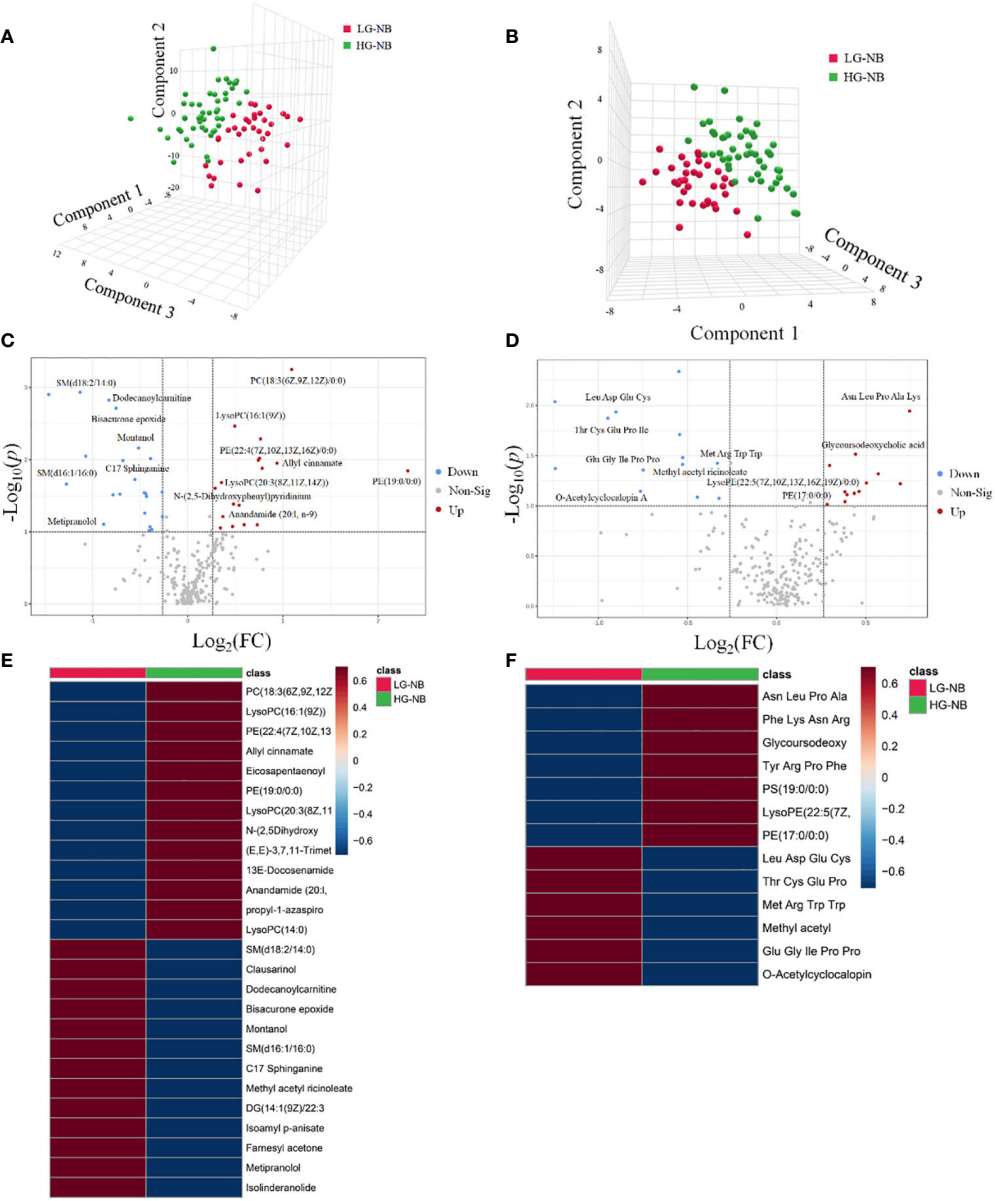
The plasma metabolomics analysis between HG-NB and LG-NB. The PLS-DA results of HG-NB and LG-NB in **(A)** positive mode and **(B)** negative mode. The volcano plot of metabolite of HG-NB and LG-NB in **(C)** positive mode and **(D)** negative mode. The heatmap shows clear distinction of metabolites between HG-NB and LG-NB in **(E)** positive mode and **(F)** negative mode.

The principal component analysis (PCA) plot demonstrates the robustness of our study by revealing distinct clustering patterns among QC samples in both positive and negative modes, as depicted in **Supplementary Figure S1**. In order to visually depict the metabolic distinctions between LG-NB and HG-NB, a cluster analysis was performed on the plasma metabolites of NB based on compound correlations and presented in the form of a heatmap (**Supplementary Figure S2**), illustrating the dissimilarities between these two groups, effectively. In order to gain insights into the metabolomics of HG-NB and LG-NB, a preliminary PLS-DA was conducted to compare HG-NB and LG-NB in both positive mode (**Figure 1A**) and negative mode (**Figure 1B**). The volcano plots depict the metabolites observed in both the HG-NB and LG-NB groups, represented in positive and negative modes (**Figures 1C, D**). Plasma metabolites exhibiting a fold change > 1.2 (fold change < 0.83) and a statistical significance of *P* < 0.05 in the volcano plot were identified as significantly altered metabolites. Therefore, a total of 26 metabolites exhibited significant changes in positive mode, comprising 13 up-regulated and 13 down-regulated metabolites (**Table 1**). In negative mode, 13 differential compounds were identified, including 7 up-regulated compounds and 6 down-regulated compounds (**Table 2**). Furthermore, in order to further visualize the differential metabolites, heatmaps of differential compounds were drawn according to the correlation of differential compounds (**Figures 1E, F**). The figure illustrates the up-regulation of compounds such as PC(18:3(6z,9z,12Z)/0:0) and the down-regulation of metabolites like SM(d18:2/14:0), indicating a significant correlation between these compounds and HG-NB. Consequently, a total of 39 differential metabolites were identified in the metabolomics analysis, highlighting substantial distinctions between HG-NB and LG-NB.

**Table 1:** Differential expressed metabolites in HG-NB vs. LG-NB in positive mode.

NO.	Metabolites	Mass-to-Charge Ratio	Retention Time(min)	VIP Value	Fold Change	*P* Value	Regu-lation
1	PC(18:3(6Z,9Z,12Z)/0:0)	517.3162	12.05	2.75	2.13	0.0005	Up
2	SM(d18:2/14:0)	672.5198	26.35	2.60	0.46	0.0012	Down
3	Clausarinol	414.204	12.22	2.59	0.36	0.0013	Down
4	Dodecanoylcarnitine	343.2720	10.84	2.55	0.56	0.0015	Down
5	Bisacurone epoxide	285.1939	8.9	2.49	0.59	0.0020	Down
6	LysoPC(16:1(9Z))	493.3165	12.2	2.36	1.41	0.0034	Up
7	Montanol	352.2607	18.27	2.19	0.70	0.0069	Down
8	SM(d16:1/16:0)	674.5355	29.44	2.12	0.48	0.0090	Down
9	PE(22:4(7Z,10Z,13Z,16Z)/0:0)	529.3164	14.88	2.10	1.69	0.0096	Up
10	Allyl cinnamate	188.0837	8.64	2.06	1.92	0.0112	Up
11	1-(5Z,8Z,11Z,14Z,17Z-Eicosapentaenoyl)-sn-glycero -3-phosphocholine	541.3162	11.98	2.01	1.72	0.0132	Up
12	PE(19:0/0:0)	495.3322	13.51	2.00	4.96	0.0143	Up
13	C17 Sphinganine	287.2096	9.2	1.91	0.68	0.0189	Down
14	LysoPC(20:3(8Z,11Z,14Z))	545.3473	13.8	1.88	1.28	0.02080	Up
15	N-(2,5Dihydroxyphenyl) pyridinium	187.0634	7.94	1.83	1.22	0.0249	Up
16	Methyl acetyl ricinoleate	354.2769	20.21	1.78	0.73	0.02873	Down
17	DG(14:1(9Z)/22:3 (10Z,13Z,16Z)/0:0)[iso2]	638.4879	38.37	1.77	0.614	0.0301	Down
18	Isoamyl p-anisate	222.1231	9.19	1.75	0.74	0.0324	Down
19	(E,E)-3,7,11-Trimethyl-2,6, 10-dodecatrienyl heptanoate	334.287	20.49	1.65	1.45	0.0409	Up
20	13E-Docosenamide	337.3344	29.58	1.53	1.29	0.016	Up
21	Farnesyl acetone	262.2297	20.33	1.53	0.83	0.0420	Down
22	Metipranolol	309.1937	9.13	1.44	0.54	0.0420	Down
23	Anandamide (20:l, n-9)	375.3111	19.5	1.43	1.51	0.0431	Up
24	(2R,6R,7S,8S)-7-Ethyl-2-propyl-1-azaspiro[5.5] undecan-8-ol	239.225	27.72	1.41	1.39	0.0446	Up
25	LysoPC(14:0)	467.3009	11.61	1.40	1.27	0.0385	Up
26	Isolinderanolide	336.2662	19.73	1.38	0.77	0.0331	Down

**Table 2:** Differential expressed metabolites in HG-NB vs. LG-NB in negative mode.

NO.	Metabolites	Mass-to-Charge Ratio	Retention Time(min)	VIP Value	Fold Change	*P* Value	Regu- lation
1	Asn Leu Pro Ala Lys	587.3225	12.01	2.36	1.676	0.0114	Up
2	Leu Asp Glu Cys	478.1716	8.48	2.36	0.5354	0.0116	Down
3	Thr Cys Glu Pro Ile	561.2453	8.75	2.31	0.5189	0.0134	Down
4	Met Arg Trp Trp	677.3127	13.51	1.95	0.7942	0.0376	Down
5	Methyl acetyl ricinoleate	400.2838	20.17	1.94	0.6938	0.0387	Down
6	Glu Gly Ile Pro Pro	511.2616	9.52	1.89	0.5952	0.0440	Down
7	Phe Lys Asn Arg	563.3227	12.00	1.78	1.4168	0.0591	Up
8	Glycoursodeoxycholic acid	449.3154	10.55	1.77	1.6163	0.0601	Up
9	O-Acetylcyclocalopin A	384.1437	9.06	1.70	0.5888	0.0715	Down
10	Tyr Arg Pro Phe	581.2945	11.71	1.69	1.3034	0.0724	Up
11	PS(19:0/0:0)	539.3229	12.21	1.68	1.3518	0.0747	Up
12	LysoPE(22:5(7Z,10Z,13Z,16Z,19Z)/0:0)	527.302	13.77	1.67	1.3143	0.0774	Up
13	PE(17:0/0:0)	513.3074	11.64	1.57	1.2168	0.0967	Up

### The altered pathways and biomarkers between HG-NB and LG-NB based on metabonomics approach

**Figure 2:**
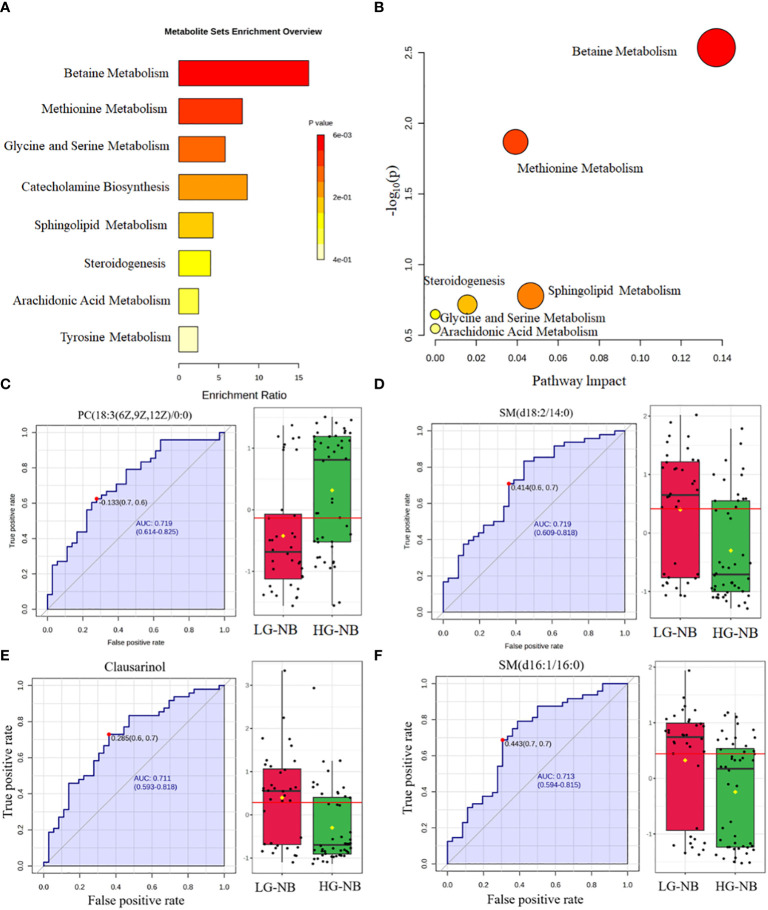
The altered pathways and biomarkers in metabolomics. **(A)** The enrichment analysis of differential metabolism revealed various metabolic changes between HG-NB and LG-NB. **(B)** The pathway analysis revealed significant abnormalities in the pathways between HG-NB and LG-NB. The representative metabolic biomarker ROC curve and boxplot of **(C)** PC (18:3(6z,9z,12Z)/0:0), **(D)** SM(d18:2/14:0), **(E)** Clausarinol and **(F)** SM(d16:1/16:0).

In order to identify abnormal metabolic pathways based on the discovery of abnormal metabolites, we performed enrichment analysis and pathway analysis. Based on the 39 most significantly altered metabolites, our enrichment analysis revealed that betaine metabolism, methionine metabolism, glycine and serine metabolism, catecholamine biosynthesis, sphingolipid metabolism, steroidogenesis, arachidonic acid metabolism, and tyrosine metabolism were enriched. (**Figure 2A**). Pathway analysis was conducted to further explore potential aberrant metabolic pathways and visualize the findings. Those results revealed significant alterations in betaine metabolism, methionine metabolism, sphingolipid metabolism, steroidogenesis, glycine and serine metabolism, as well as arachidonic acid metabolism (**Figure 2B**). Consequently, enrichment analysis revealed 8 significantly altered metabolic pathways, while pathway analysis identified 6 additional significantly altered metabolic pathways, thereby enhancing our comprehension of the aberrant NB pathway network.

To investigate plasma biomarkers associated with HG-NB in metabolomics and propose a non-invasive approach for risk stratification of NB, we conducted receiver operating characteristic (ROC) curve analysis on differential metabolites. The iconic biomarkers PC(18:3(6z,9z,12Z)/0:0), SM (d18:2/14:0), Clausarinol and SM(d16:1/16:0) were identified in this study (**Figures 2C–F**). The area under the curve (AUC) of the ROC analysis for all biomarkers exceeded 0.7, suggesting that these metabolites have potential as biomarkers for HG-NB. In summary, our enrichment analysis revealed 10 altered metabolic pathways, while pathway analysis identified 6 altered metabolic pathways. Additionally, 4 biomarkers were discovered through our comprehensive biomarker analysis. These findings provide valuable insights into understanding the aberrant NB pathway network and offer potential targets for targeted therapy.

### Transcriptomics analysis uncovers the abnormal expression gene between HG-NB and LG-NB

**Figure 3:**
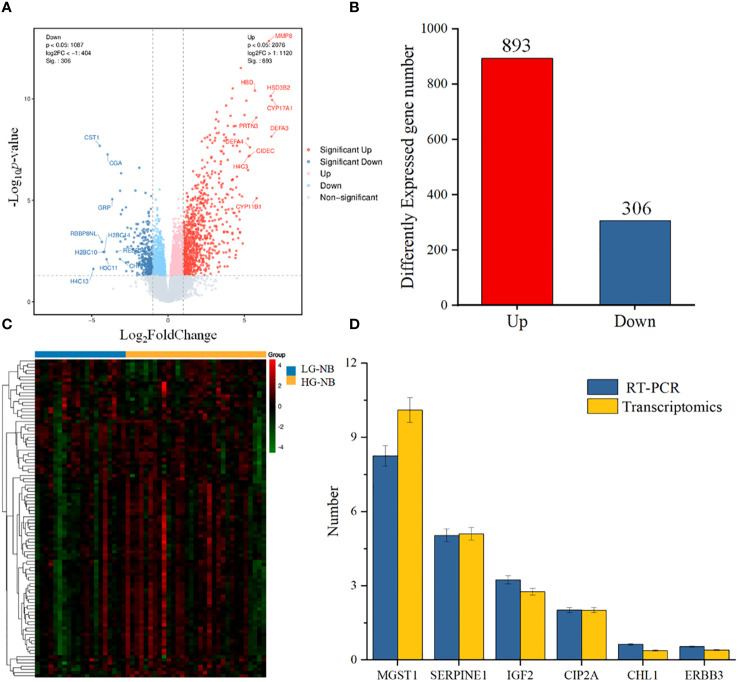
The NB tissue transcriptomics and validation. **(A)** The volcano plot shows differentially expressed genes between HG-NB and LG-NB. **(B)** Number of differentially expressed genes of HG-NB compared to LG-NB. **(C)** The heatmap shows segregation of HG-NB and LG-NB based on transcriptomics analysis. **(D)** Expression trends of genes in RT-PCR were consistent with transcriptomics results.

To further investigate the disparities between HG-NB and LG-NB, we conducted transcriptomics analysis on 31 HG-NB tissues and 20 LG-NB tissues. The comprehensive outcomes of total RNA concentration, A_260_/A_280_ ratio, A_260_/A_230_ ratio, 28S/18S ratio, and RNA integrity number for the extracted samples are presented in **Supplementary Table S3**. All the RNA integrity number values obtained in this study exceeded 7. The preprocessing results of sequencing data quality revealed that RawBases values ranged from 6.49G to 7.76G per sample, CleanBases values ranged from 6.00G to 7.22G per sample, and the percentage of Q30 bases varied from 92.59% to 95.18% across all samples. The GC content of each sample ranged from 47.87% to 49.35% (**Supplementary Table S4**). In conjunction with the total number of mRNAs detected in the samples (**Supplementary Figure S3**) and FPKM values (**Supplementary Figure S4**), it can be inferred that the RNA quality of both groups adhered to established standards, rendering them suitable for subsequent analyses. To visually represent the transcriptomics disparities between LG-NB and HG-NB, we conducted hierarchical clustering analysis based on RNA correlation using tissue RNA samples from NB. The results were presented in a heatmap (**Supplementary Figure S5**), highlighting the evident differences between the two groups. Differential genes were identified as NB tissue RNAs with *P* < 0.05 and |log_2_ (fold change)| > 1 in the volcano plot (**Figure 3A**). A total of 1,199 differentially expressed genes were identified, comprising 893 up-regulated genes and 306 down-regulated genes (**Figure 3B**). The up-regulated and down-regulated genes in HG-NB and LG-NB tissues are presented in **Tables 3**, **4**, respectively. A cluster analysis heatmap (**Figure 3C**) was employed to visually depict the top 100 differentially expressed genes, facilitating a more comprehensive understanding of the distinctions between HG-NB and LG-NB groups, thereby highlighting their significant differences. Based on the transcriptome results and relevant literature on NB (22–27), we selected 6 reported differentially expressed genes, namely *MGST1*, *SERPINE1*, *IGF2*, *CIP2A*, *CHL1*, and *ERBB3* for RT-PCR validation. Our RT-PCR results demonstrated that the relative expressions of *MGST1*, *SERPINE1*, *IGF2*, and *CIP2A* were significantly increased in HG-NB compared to LG-NB, while the relative expressions of *CHL1* and *ERBB3* were significantly decreased in HG-NB compared to LG-NB. Importantly, our transcriptomics findings were consistent with the RT-PCR results which further validate their reliability (**Figure 3D**). In summary, our transcriptomics analysis revealed significant differences between HG-NB and LG-NB.

**Table 3:** The top 20 genes significantly up-regulated in HG-NB vs. LG-NB.

NO.	Gene	Description	Fold Change	*P* value
1	*CYP17A1*	cytochrome P450 family 17 subfamily A member 1	112.848	1.11E-10
2	*DEFA3*	defensin alpha 3	109.644	7.18E-09
3	*HSD3B2*	hydroxy-delta-5-steroid dehydrogenase, 3 beta- and steroid delta-isomerase 2	105.903	7.18E-11
4	*MMP8*	matrix metallopeptidase 8	98.087	1.41E-13
5	*CYP11B1*	cytochrome P450 family 11 subfamily B member 1	55.990	7.82E-06
6	*PRTN3*	proteinase 3	55.314	8.29E-10
7	*HBD*	hemoglobin subunit delta	51.879	3.91E-11
8	*DEFA4*	defensin alpha 4	41.410	2.42E-08
9	*CIDEC*	cell death inducing DFFA like effector c	40.741	6.28E-08
10	*H4C3*	H4 clustered histone 3	38.945	6.70E-08
11	*CEACAM8*	CEA cell adhesion molecule 8	37.945	3.24E-07
12	*MS4A3*	membrane spanning 4-domains A3	37.691	9.09E-09
13	*WDR72*	WD repeat domain 72	35.055	1.24E-10
14	*PLIN1*	perilipin 1	32.315	6.25E-10
15	*H4C2*	H4 clustered histone 2	29.826	0.001338
16	*TRARG1*	trafficking regulator of GLUT4 (SLC2A4) 1	28.888	6.38E-06
17	*PCOLCE2*	procollagen C-endopeptidase enhancer 2	27.376	3.01E-12
18	*SULT2A1*	sulfotransferase family 2A member 1	26.360	0.000365
19	*MC2R*	melanocortin 2 receptor	26.289	0.000393
20	*C14orf180*	chromosome 14 open reading frame 180	26.137	3.88E-08

**Table 4:** The top 20 genes significantly down-regulated in HG-NB vs. LG-NB.

NO.	Gene	Description	Fold Change	*P* value
1	*HES3*	hes family bHLH transcription factor 3	0.150	0.030653
2	*H2BC13*	H2B clustered histone 13	0.149	0.011684
3	*UTS2*	urotensin 2	0.148	2.22E-05
4	*PAX3*	paired box 3	0.144	0.000821
5	*LOC102724265*	uncharacterized LOC102724265	0.135	0.000599
6	*NTSR2*	neurotensin receptor 2	0.125	2.98E-05
7	*UCN3*	urocortin 3	0.119	4.64E-07
8	*PKLR*	pyruvate kinase L/R	0.118	4.80E-05
9	*POU5F2*	POU domain class 5, transcription factor 2	0.115	0.00107
10	*ADCYAP1*	adenylate cyclase activating polypeptide 1	0.113	3.36E-06
11	*CHRNB3*	cholinergic receptor nicotinic beta 3 subunit	0.113	0.007814
12	*RESP18*	regulated endocrine specific protein 18	0.098	0.003363
13	*GRP*	gastrin releasing peptide	0.079	8.77E-06
14	*CGA*	glycoprotein hormones, alpha polypeptide	0.064	5.46E-08
15	*H3C11*	H3 clustered histone 11	0.061	0.007993
16	*H2BC14*	H2B clustered histone 14	0.055	0.003496
17	*H2BC10*	H2B clustered histone 10	0.053	0.003473
18	*RBBP8NL*	RBBP8 N-terminal like	0.050	0.001128
19	*CST1*	cystatin SN	0.045	2.06E-08
20	*H4C13*	H4 clustered histone 13	0.034	0.024042

### KEGG and GO analysis between HG-NB and LG-NB in transcriptomics

**Figure 4:**
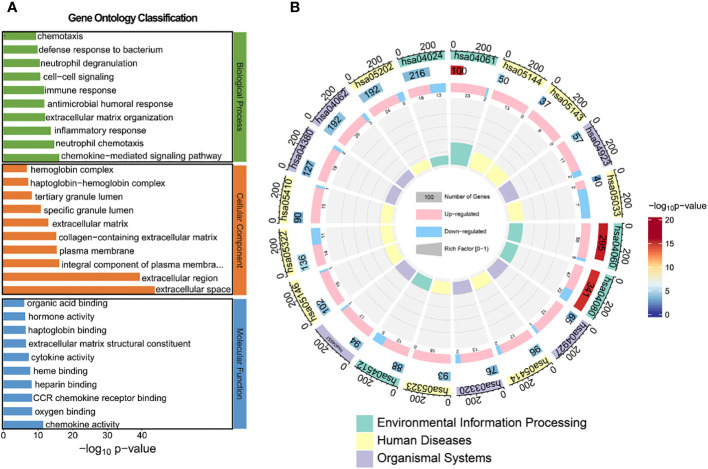
The GO and KEGG analysis between HG-NB and LG-NB in transcriptomics. **(A)** GO analysis of biological processes, molecular functions and cellular components organization of up- and down-regulated genes between HG-NB and LG-NB. **(B)** KEGG analyzes up- and down-regulated genes from 3 aspects of environmental information, human diseases and organismal systems between HG-NB and LG-NB.

To identify aberrant pathways based on differential gene expression, we conducted GO analysis and KEGG analysis. Subsequently, GO annotation analysis was performed to elucidate the metabolic pathways associated with these differentially expressed genes and infer their potential biological functions. The obtained differential genes were subjected to GO analysis in order to elucidate the metabolic pathways associated with these genes and infer their potential biological functions, as depicted in **Figures 4A** and **Supplementary Figure S6**. In terms of biological processes, the top three regulated expressions comprised the chemokine-mediated signaling pathway, neutrophil chemotaxis, and inflammatory response. Regarding cellular components, the top three significantly regulated expressions were extracellular space, extracellular region, and integral component of plasma membrane. Concerning molecular function, the top three significantly up-regulated expressions included chemokine activity, oxygen binding, and CCR chemokine receptor binding. We further conducted KEGG prediction analysis and observed that the neuroactive ligand-receptor interaction, cytokine-cytokine receptor interaction, and cAMP signaling pathway exhibited the three most pronounced alterations. This suggests an alternative perspective on the biological functions of HG-NB (**Figure 4B**, **Supplementary Figure S7**). The annotation table for each pathway in **Figure 4B** is presented in **Supplementary Table S5**. Consequently, the molecular mechanisms of HG-NB that impact prognosis include chemokine-mediated signaling pathways, neutrophil chemotaxis, inflammatory responses, extracellular space and extracellular region, integral components of the plasma membrane, chemokine activity, oxygen binding, CCR chemokine receptor binding, neuroactive ligand-receptor interactions, cytokine-cytokine receptor interactions, and cAMP signaling pathways. Therefore, employing transcriptomics methods has revealed multiple biological functional differences between HG-NB and LG-NB, which is expected to provide a theoretical foundation for exploring the molecular mechanisms underlying HG-NB.

### Integrated transcriptomics and metabolomics analyses between HG-NB and LG-NB

**Figure 5:**
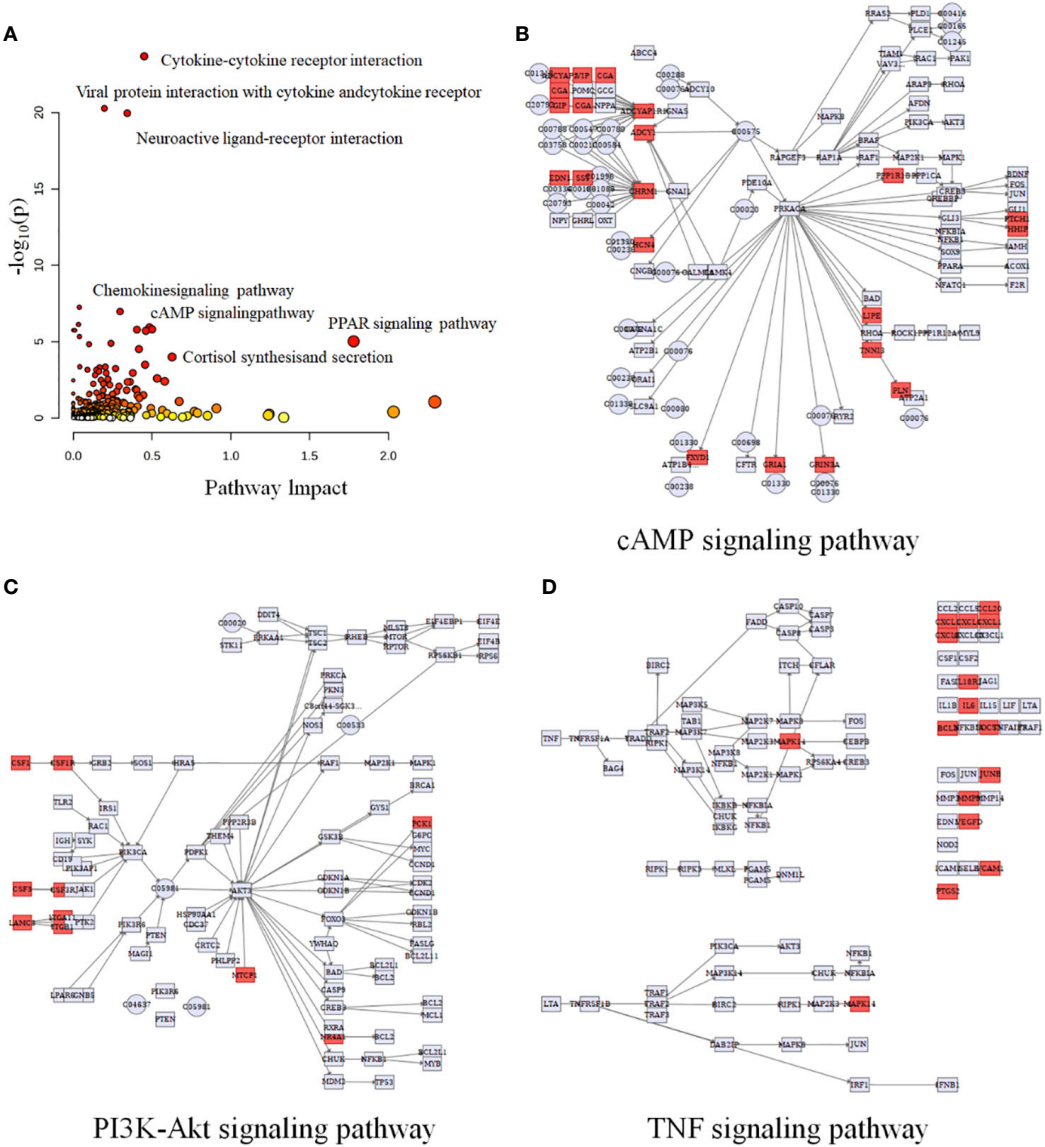
Integrated transcriptomics and metabolomics analysis of NB metabolic pathways. **(A)** Joint-pathway analysis of differential pathway between HG-NB and LG-NB. **(B)** The cAMP signaling pathway, **(C)** the PI3K-Akt signaling pathway and **(D)** the TNF signaling pathway with altered significantly genes in HG-NB compared to LG-NB. Significant overexpression in red, and no significant changes in grey.

Multi-omics studies employ integrative research approaches to comprehensively integrate data and regulatory relationships across multiple levels, enabling a multifaceted exploration of disease mechanisms (28). To systematically investigate NB, we employed joint-pathway analysis to establish connections between metabolites and genes through shared metabolic pathways. Through an integrated analysis of transcriptomics and metabolomics data, we identified 10 significantly altered pathways (**Table 5**). The dysregulated pathways, such as cytokine-cytokine receptor interaction, viral protein interaction with cytokine and cytokine receptor, neuroactive ligand-receptor interaction, etc., exhibiting *P* values < 0.05, were visually represented in **Figure 5A**. The cAMP signaling pathway, as depicted in **Figure 5B**, exhibited statistical significance with *P* values < 0.05 and an impact coefficient of 0.48. Notably, this pathway encompassed a set of significantly altered genes including *CGA*, *ADRB1*, *GIP*, *ADCY1*, *SST*, *FFAR2*, *HCN4*, *PPP1R1B*, *PTCH1*, *HHIP*, *LIPE*, *TNNI3*, *PLN*, *FXYD1*, *GRIA1*, and *GRIN3A*. As depicted in **Figure 5C**, the PI3K-Akt signaling pathway exhibited alterations in the expression levels of *CSF1*, *CSF1R*, *PCK1*, *IL6*, *CHAD* and *TCL1A* between HG-NB and LG-NB. **Figure 5D** shows that in TNF signaling pathway, *MAPK13*, *CCL20*, *CXCL1*, *IL18R1*, *BCL3*, *SOCS3*, *JUNB*, *MMP9*, *VEGFC*, *VCAM1*, and *PTGS2* were altered in HG-NB. Therefore, through integrated metabolomics and transcriptomics analysis, we identified significant alterations in the cAMP signaling pathway, PI3K-Akt signaling pathway, and TNF signaling pathway in MNA NB. These findings provide a solid theoretical foundation for future therapeutic strategies targeting HG-NB.

**Table 5:** Differential metabolic pathways based on joint-pathway analysis.

NO	Pathway name		Match status	*P* value		Impact
1	Cytokine-cytokine receptor interaction		64/294	<0.001		0.45
2	Viral protein interaction with cytokine and cytokine receptor		35/100	<0.001		0.20
3	Neuroactive ligand-receptor interaction		69/392	<0.001		0.34
4	Chemokine signaling pathway		29/194	<0.001		0.30
5	PI3K-Akt signaling pathway		33/358	<0.001		0.17
6	cAMP signaling pathway		31/241	<0.001		0.48
7	ECM-receptor interaction		17/89	<0.001		0.5
8	PPAR signaling pathway		15/81	<0.001		1.8
9	Cortisol synthesis and secretion		13/77	<0.001		0.63
10	TNF signaling pathway		15/112	<0.001		0.04

### Classification of NB with selected transcriptome candidate biomarkers

To enhance the low rate of early diagnosis of HG-NB, a more efficient risk diagnostic model was established as a complementary approach to existing methods. The 6 candidate genes identified through transcriptomics and literature were subjected to RT-PCR analysis in order to identify biomarkers suitable for diagnosis (**Supplementary Table S6**).

**Figure 6:**
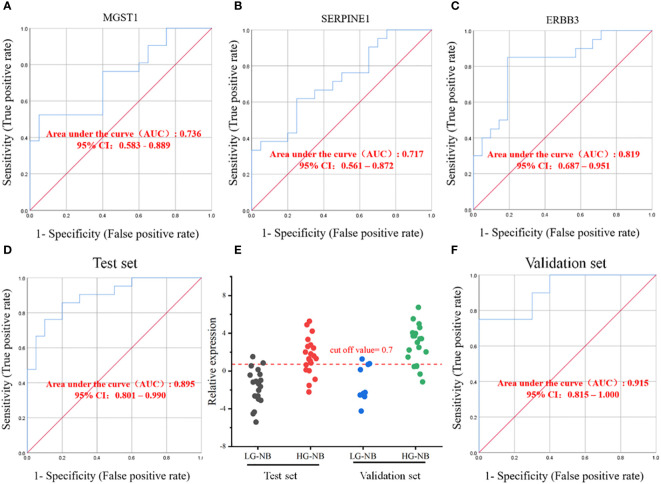
Establishment of HG-NB early diagnosis model. The ROC curves for biomarkers **(A)** *MGSTI*, **(B)** *SERPINE1* and **(C)** *ERBB3*. **(D)** The ROC curve in the test set. **(E)** The prediction accuracies by the *MGSTI*, *SERPINE1*, and *ERBB3* in test set and validation set are compared between HG-NB and LG-NB. **(F)** The ROC curve in the validation set.

The results for individual candidate genes were calculated using equation 2^-ΔΔCt^, and subsequently the sensitivity and specificity were determined. However, the findings revealed that the areas under the ROC curve of *MGST1*, *SERPINE1* and *ERBB3* were 0.736, 0.717, and 0.819 respectively, indicating a limited detection performance of these individual biomarkers (**Figures 6A–C**). The ROC curves for the remaining 3 biomarkers are presented in **Supplementary Figure S8**, with none of them achieving an AUC greater than 0.7. Consequently, a diagnostic model integrating *MGST1*, *SERPINE1* and *ERBB3* three biomarkers was established through logistic regression analysis to obtain the regression equation *Y* = -3.393 + 0.436 *X*_1_ (*MGST1*) + 0.491 *X*_2_ (*SERPINE1*) - 0.498 *X*_3_ (*ERBB3*). In the test set, the diagnostic model exhibited a sensitivity of 71% and specificity of 90%. The ROC analysis yielded an area under the curve (AUC) value of 0.895 (**Figure 6D**), with a cutoff value set at 0.7 (**Figure 6E**). In the validation set, the diagnostic model demonstrated a sensitivity of 75% and specificity of 80%, while achieving an AUC value of 0.915 in ROC analysis (**Figure 6F**), thus confirming its efficacy. Therefore, *MGST1*, *SERPINE1* and *ERBB3* represent viable biomarkers that can be utilized in combination as a diagnostic model for predicting the plasma risk classification of NB. This approach holds promise for non-invasive and cost-effective detection of NB at an early stage.

## Discussion

The INSS staging system is a surgical-pathological staging system that is based on the site of origin and metastasis of NB. Originally proposed in 1988 and revised in 1993, it serves as a crucial tool for risk assessment and subsequent management of NB (29, 30). Except for stage 4S, the risk score of INSS patients increases progressively from stage 1 to stage 4 (31). However, the early prediction of NB and the lack of effective therapeutic targets remain significant challenges in current research. Therefore, we conducted a comprehensive analysis of the disparities between HG-NB and LG-NB by integrating metabolomics and transcriptomics. Our findings revealed significant distinctions in the cAMP signaling pathway, PI3K-Akt signaling pathway, and TNF signaling pathway. Furthermore, we identified 3 biomarkers with notable variances (*MGST1*, *SERPINE1* and *REBB3*) and developed a diagnostic model. These advancements hold immense significance for the timely detection of HG-NB.

By integrating metabolomics and transcriptomics analysis, we have identified significant disparities in the cAMP signaling pathway between LG-NB and HG-NB. Initially discovered over 60 years ago, cAMP is a extensively investigated second messenger implicated in diverse cellular processes, encompassing growth, differentiation, and gene transcription (32). Adenylyl cyclase is a membrane-bound enzyme responsible for the conversion of adenosine triphosphate into cAMP. cAMP, in turn, exerts its effects on four effector proteins: exchange protein activated by cAMP, cyclic-nucleotide gated ion channels, Popeye proteins, and the cAMP-dependent PKA pathway (33–35). Previous studies have demonstrated the pivotal role of cAMP in various malignancies, including prostate cancer, ovarian cancer, and lung cancer (36–38). *ADCY1* serves as a pivotal regulator of the cAMP signaling pathway and is accountable for catalyzing ATP to cAMP. In the investigation conducted by Zou et al., it was highlighted that *ADCY1* holds immense significance as a novel biomarker in predicting drug resistance among patients with lung cancer (38). Our study also revealed a significant disparity in the transcriptomics of *ADCY1* (Fold change = 0.45). Therefore, further investigation is warranted to elucidate the potential impact of *ADCY1* on the INSS grade of NB through modulation of the cAMP signaling pathway. Furthermore, a robust correlation between cAMP and cell death was observed. The functional mitochondrial cAMP pathway in neonatal and adult cardiomyocytes plays a pivotal role in regulating cell death, with activation of this pathway exerting an inhibitory effect on apoptotic processes (39). Moreover, the induction of tumor cell death has been widely acknowledged as an effective therapeutic strategy (40). Therefore, targeting the cAMP signaling pathway to modulate cellular apoptosis may represent a novel avenue for improving prognosis.

The combined analysis of metabolomics and transcriptomics revealed that the PI3K-Akt signaling pathway exhibited significant alterations. The PI3K-Akt signaling pathway is aberrantly activated during the occurrence and progression of certain cancers. The two most extensively elucidated mechanisms underlying PI3K-Akt activation in human cancer involve receptor tyrosine kinase stimulation and somatic mutations in specific components of signaling pathways (41). Augmentation and facilitation of the PI3K-Akt pathway may exert a detrimental impact on cancer therapy; hence, inhibition of PI3K could impede cancer development (42). The PI3K-Akt signaling pathway was found to be significantly dysregulated in HG-NB, which is associated with tumor growth, angiogenesis, and survival. Loss of function of the tumor suppressor gene *PTEN* is a common event in human tumors that leads to aberrant activation of the PI3K/Akt pathway (43, 44). Furthermore, the pivotal role of the PI3K-Akt signaling pathway in tumor resistance has been well-established. The regulatory effect of berberine on cell death across various cancer types through modulation of the PI3K-Akt signaling pathway has also been elucidated (45, 46). Wu et al. demonstrated that the activation of the PI3K-Akt signaling pathway can induce cell death by suppressing autophagy, thereby providing novel insights into the intricate relationship between the PI3K-AKT signaling pathway and cellular demise (47). Therefore, a comprehensive investigation into the underlying mechanisms governing cell death mediated by the PI3K-Akt signaling pathway will contribute to unraveling disease pathogenesis and identifying potential targets for clinical intervention in HG-NB.

Through the integration of metabolomics and transcriptomics studies, we have identified significant alterations in the TNF signaling pathway. Tumor necrosis factor (TNF) is a multifunctional cytokine with immunological effects, playing a pivotal role in both adaptive and innate immunity as well as the homeostasis of immune cells. Its action and production are temporally and spatially regulated (48). Activated macrophages, T lymphocytes, and natural killer cells that secrete TNF are distributed systemically via the bloodstream, encompassing various anatomical regions including the musculoskeletal system (49). Moreover, it has been proposed that TNF is implicated in tumor angiogenesis and cell death, thereby facilitating tumor advancement and metastasis (50, 51). The activation of the TNF signaling pathway can induce the expression and activation of a diverse array of downstream molecules, including nuclear factor κB and p38 mitogen-activated protein kinase (52, 53). Subsequent activation of these molecules exerts regulatory control over various biological processes, such as cell death. The TNF signaling pathway has been confirmed to be intricately associated with various diseases, including prostate cancer, breast cancer, and gastric cancer (54, 55). However, further investigations are warranted to elucidate the underlying mechanisms of the TNF signaling pathway in NB.

## Conclusions

In this study, a total of 84 clinical plasma samples and 51 clinical NB tissue samples were analyzed, leading to the identification of 1,199 differential genes and 39 differential metabolites. The metabolomics and transcriptomics characteristics of HG-NB patients were elucidated, followed by a comprehensive network analysis. Furthermore, significant differences in key signaling pathways including cAMP signaling pathway, PI3K-Akt signaling pathway, and TNF signaling pathway were observed between HG-NB and LG-NB. Subsequently, a risk stratification risk diagnostic model for HG-NB was developed based on the combination of *MGST1*, *SERPINE1*, and *ERBB3*. The area under the ROC curve was determined to be 0.915, while the sensitivity and specificity were found to be 75.0% and 80.0%, respectively, indicating the potential of the risk diagnostic model for early detection of HG-NB as well as its future therapeutic implications. The limited sample size of this study was inadequate, and the diagnostic model we constructed could not be clinically validated. In future studies, our aim is to increase the sample size, identify potential biomarkers, explore effective therapeutic targets, and enhance patient outcomes. In summary, a comprehensive analysis integrating metabolomics and transcriptomics revealed a dysregulated network, leading to the development of a diagnostic model for HG-NB.

## Data availability statement

The datasets presented in this study can be found in online repositories. The names of the repository/repositories and accession number(s) can be found below: https://www.ncbi.nlm.nih.gov/, PRJNA884866.

## Ethics statement

This study was reviewed and approved by the committees of Henan Children’s Hospital. The studies were conducted in accordance with the local legislation and institutional requirements. Written informed consent for participation in this study was provided by the participants’ legal guardians/next of kin.

## Author contributions

BD: Methodology, Writing – review & editing. WZ: Writing – original draft. MZ: Data curation, Writing – review & editing. MS: Data curation, Writing – review & editing. MH: Data curation, Writing – review & editing. MY: Software, Writing – review & editing. JS: Software, Writing – review & editing. XZ: Writing – review & editing, Methodology.
