# Supplementary material for: Metabolomics-transcriptomics joint analysis: unveiling the dysregulated cell death network and developing a diagnostic model for high-grade neuroblastoma
Source: Front Immunol. 2024 Jan 4;14:1345734. doi: 10.3389/fimmu.2023.1345734 (PMC10794662; doi:10.3389/fimmu.2023.1345734)
Supplement: Supplementary file 1 [file DataSheet_1.docx]

**Supporting information**

**Metabolomics-transcriptomics joint analysis: Unveiling the dysregulated network and developing a diagnostic model for high-grade neuroblastoma**

Wancun Zhang^1,2,3^, Mengxin Zhang^1^, Meng Sun^3^, Minghui Hu^1^, Muchun Yu^2^, Jushan Sun^1^, Xianwei Zhang^1,^*, Bang Du ^1,2,3,^*

^1^Health Commission of Henan Province Key Laboratory for Precision Diagnosis and Treatment of Pediatric Tumor, Children’s Hospital Affiliated to Zhengzhou University, Zhengzhou 450018, China.

^2^Henan International Joint Laboratory for Prevention and Treatment of Pediatric Disease, Children’s Hospital Affiliated to Zhengzhou University, Zhengzhou 450018, China.

^3^Henan Key Laboratory of Children’s Genetics and Metabolic Diseases, Children’s Hospital Affiliated to Zhengzhou University, Zhengzhou 450018, China.

^*^Corresponding author E-mail addresses: [dubang0820@163.com (Bang](mailto:dubang0820@163.com%20(Bang) Du); [zhangxw956658@126.com](mailto:zhangxw956658@126.com) (Xianwei Zhang).

**Table S1** **Baseline Characteristics of HG-NB *vs.* LG-NB in metabolomics**

|  | HG-NB | LG-NB | *P* Value |
| --- | --- | --- | --- |
| Number | 48 | 36 | / |
| Age (month) | 41.93±33.92 | 37.14±35.06 | 0.533 |
| Male (%) | 0.625 | 0.50 | 0.252 |
| *MYCN* (%) | 39.58 | 41.67 | 0.847 |
| Radiological risk factors (%) | 97.92 | 30.56 | <0.001 |
| Gross tumor volume (cm^3^) | 173.15±226.45 | 90.79±130.60 | 0.018 |

**Table S2** **Baseline Characteristics of HG-NB *vs.* LG-NB in transcriptomics**

|  | HG-NB | LG-NB | *P* Value |
| --- | --- | --- | --- |
| Number | 31 | 20 | / |
| Age (month) | 42.52±35.25 | 44.41±42.41 | 0.523 |
| Male (%) | 67.74 | 45.0 | 0.107 |
| *MYCN* (%) | 38.71 | 30.0 | 0.525 |
| Radiological risk factors (%) | 93.55 | 15.0 | <0.001 |
| Gross tumor volume (cm^3^) | 171.75±194.52 | 66.48±63.85 | 0.009 |


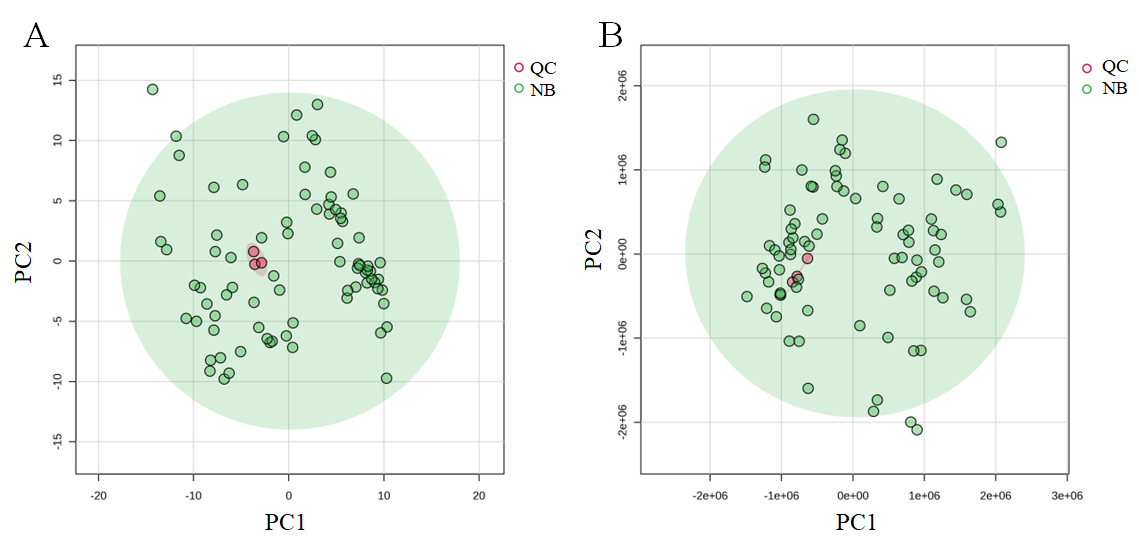


**Figure S1** PCA plot between QC samples and metabolomics samples. (A) QC samples clumped together in positive mode. (B) QC samples clumped together in negative mode.


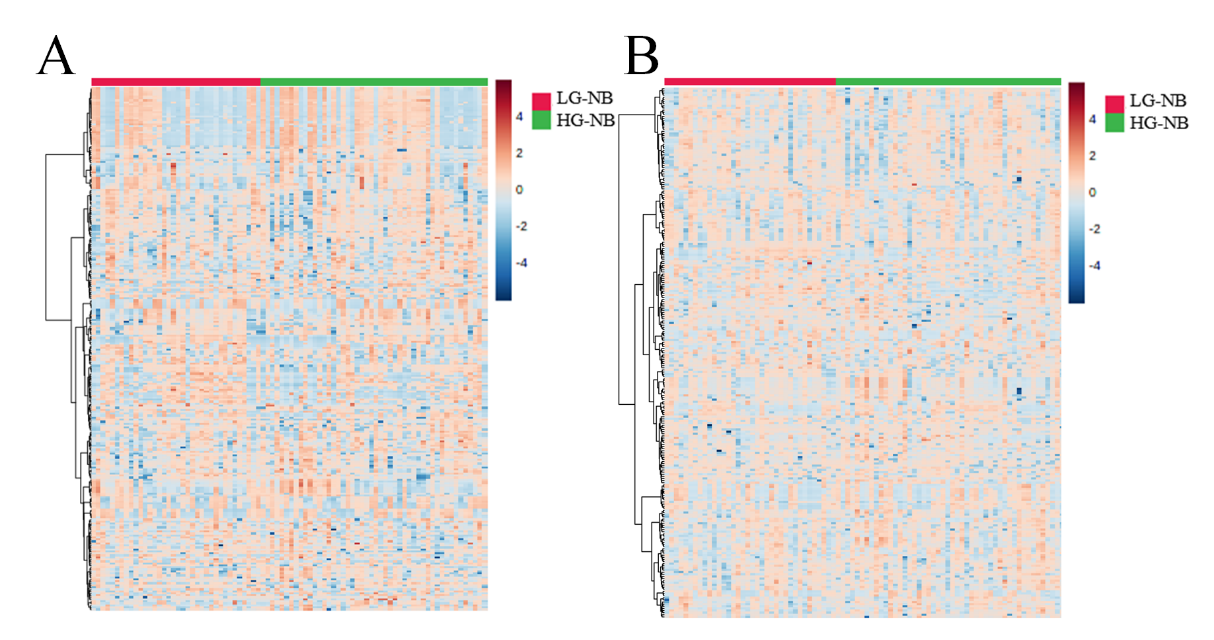


**Figure S2** Heatmap of all metabolites between HG-NB and LG-NB in metabolomics. (A) Metabolite expression in positive mode. (B) Metabolite expression in negative mode.

**Table S3** **Transcriptome sample quality control sheet**

| **No.** | **Concentration（μg/μL）** | **A_260/280_** | **A_260/230_** | **Volume (μL)** | **Total（μg）** | **28S/18S** | **RIN** |
| --- | --- | --- | --- | --- | --- | --- | --- |
| 1 | 0.0666 | 2.1 | 1.95 | 20 | 1.33 | 1.2 | 8.2 |
| 2 | 0.0686 | 2.09 | 1.85 | 15 | 1.03 | 1.3 | 7.5 |
| 3 | 0.0712 | 2.09 | 1.86 | 15 | 1.07 | 2.5 | 8.8 |
| 4 | 0.0712 | 2.05 | 1.83 | 14 | 1 | 1.6 | 7.5 |
| 5 | 0.0888 | 2.09 | 1.99 | 15 | 1.33 | 2.2 | 7.3 |
| 6 | 0.0928 | 2.04 | 1.51 | 15 | 1.39 | 2.2 | 7.9 |
| 7 | 0.0936 | 2.09 | 1.99 | 15 | 1.4 | 1.6 | 8.4 |
| 8 | 0.0941 | 2.09 | 2.06 | 40 | 3.76 | 1.1 | 7 |
| 9 | 0.1015 | 2.07 | 1.97 | 15 | 1.52 | 2.8 | 8 |
| 10 | 0.1027 | 2.09 | 2 | 30 | 3.08 | 1.3 | 7.3 |
| 11 | 0.1059 | 2.05 | 2.13 | 40 | 4.24 | 1.7 | 7.9 |
| 12 | 0.106 | 2.08 | 2.08 | 40 | 4.24 | 1.4 | 7.9 |
| 13 | 0.1061 | 2.03 | 1.91 | 10 | 1.06 | 1.3 | 7.4 |
| 14 | 0.1069 | 2.09 | 2.06 | 40 | 4.28 | 1.4 | 8 |
| 15 | 0.1086 | 2.07 | 2.04 | 40 | 4.34 | 1.7 | 7.4 |
| 16 | 0.1141 | 2.09 | 2.09 | 40 | 4.56 | 1.3 | 7.6 |
| 17 | 0.1156 | 2.05 | 2.12 | 40 | 4.62 | 1.2 | 7.8 |
| 18 | 0.126 | 2.06 | 2.2 | 40 | 5.04 | 1.6 | 8.5 |
| 19 | 0.1278 | 2.09 | 1.89 | 15 | 1.92 | 1.6 | 8.2 |
| 20 | 0.1348 | 2.09 | 2.06 | 40 | 5.39 | 1.5 | 7.6 |
| 21 | 0.1358 | 2.08 | 2.03 | 20 | 2.72 | 1.5 | 8.2 |
| 22 | 0.1386 | 2.07 | 2.09 | 40 | 5.54 | 1.6 | 7.2 |
| 23 | 0.1525 | 2.08 | 2.11 | 40 | 6.1 | 2.3 | 7.9 |
| 24 | 0.1547 | 2.06 | 2.16 | 40 | 6.19 | 2.1 | 8 |
| 25 | 0.1593 | 2.05 | 2.14 | 40 | 6.37 | 1.5 | 7.5 |
| 26 | 0.1729 | 2.06 | 2.15 | 40 | 6.92 | 2 | 7.5 |
| 27 | 0.1729 | 2.06 | 2.16 | 40 | 6.92 | 1 | 7.9 |
| 28 | 0.1754 | 2.04 | 2.16 | 40 | 7.02 | 1.6 | 7.3 |
| 29 | 0.178 | 2.05 | 2.13 | 40 | 7.12 | 2 | 8.9 |
| 30 | 0.1788 | 2.05 | 2.17 | 40 | 7.15 | 1.5 | 8.9 |
| 31 | 0.1797 | 2.04 | 2.16 | 40 | 7.19 | 1.7 | 8 |
| 32 | 0.1839 | 2.04 | 2.17 | 40 | 7.36 | 1.5 | 7.2 |
| 33 | 0.1898 | 2.03 | 2.15 | 40 | 7.59 | 1 | 8.1 |
| 34 | 0.2047 | 2.04 | 2.24 | 40 | 8.19 | 1.5 | 7.5 |
| 35 | 0.2153 | 2.01 | 2.19 | 40 | 8.61 | 1.7 | 8.1 |
| 36 | 0.2403 | 2.02 | 2.26 | 40 | 9.61 | 2.3 | 7.9 |
| 37 | 0.2815 | 2.04 | 2.18 | 40 | 11.26 | 1.9 | 7.2 |
| 38 | 0.282 | 2.04 | 2.17 | 40 | 11.28 | 1.7 | 7 |
| 39 | 0.2929 | 2.04 | 2.17 | 40 | 11.72 | 1.2 | 7 |
| 40 | 0.335 | 2.05 | 2.11 | 40 | 13.4 | 0.8 | 7.2 |
| 41 | 0.3374 | 1.99 | 2.22 | 40 | 13.5 | 2 | 7.9 |
| 42 | 0.3414 | 2.04 | 2.14 | 40 | 13.66 | 1.5 | 7.6 |
| 43 | 0.3706 | 2.01 | 2.23 | 40 | 14.82 | 2 | 7.5 |
| 44 | 0.396 | 2.03 | 2.19 | 40 | 15.84 | 1.8 | 7.5 |
| 45 | 0.4264 | 1.98 | 2.21 | 40 | 17.06 | 1.9 | 8.6 |
| 46 | 0.4452 | 1.98 | 2.22 | 40 | 17.81 | 1.3 | 7.1 |
| 47 | 0.4808 | 2 | 2.19 | 40 | 19.23 | 2.9 | 8.8 |
| 48 | 0.4944 | 2.04 | 2.31 | 40 | 19.78 | 2.4 | 8.1 |
| 49 | 0.499 | 1.97 | 2.22 | 40 | 19.96 | 2.2 | 8.5 |
| 50 | 0.7011 | 2.03 | 2.28 | 40 | 28.04 | 1.9 | 8.1 |
| 51 | 0.9507 | 2.01 | 2.26 | 40 | 38.03 | 2.6 | 8.3 |

**Table S4** **Summary of transcriptome sequencing data quality preprocessing results**

| **NO.** | **Raw**  **Reads** | **Raw**  **Bases** | **Clean**  **Reads** | **Clean**  **Bases** | **Valid**  **Bases** | **Q30** | **GC** |
| --- | --- | --- | --- | --- | --- | --- | --- |
| 1 | 48.93M | 7.34G | 47.98M | 6.78G | 92.39% | 93.08% | 49.63% |
| 2 | 48.91M | 7.34G | 48.00M | 6.72G | 91.66% | 94.13% | 49.83% |
| 3 | 47.47M | 7.12G | 46.46M | 6.53G | 91.73% | 92.59% | 47.87% |
| 4 | 48.14M | 7.22G | 47.24M | 6.71G | 92.93% | 94.65% | 49.20% |
| 5 | 49.63M | 7.44G | 48.70M | 6.86G | 92.09% | 93.19% | 49.79% |
| 6 | 48.15M | 7.22G | 47.32M | 6.68G | 92.45% | 93.28% | 49.80% |
| 7 | 51.59M | 7.74G | 50.68M | 7.20G | 93.08% | 94.89% | 49.19% |
| 8 | 51.12M | 7.67G | 50.26M | 7.13G | 92.95% | 94.74% | 47.93% |
| 9 | 51.53M | 7.73G | 50.48M | 7.10G | 91.84% | 92.74% | 47.92% |
| 10 | 49.37M | 7.40G | 48.44M | 6.81G | 91.92% | 93.91% | 49.15% |
| 11 | 43.28M | 6.49G | 42.55M | 6.00G | 92.40% | 94.28% | 49.12% |
| 12 | 50.49M | 7.57G | 49.65M | 7.03G | 92.87% | 94.64% | 48.35% |
| 13 | 46.48M | 6.97G | 45.54M | 6.37G | 91.38% | 93.83% | 49.58% |
| 14 | 51.36M | 7.70G | 50.39M | 7.14G | 92.63% | 95.05% | 47.46% |
| 15 | 47.04M | 7.06G | 46.16M | 6.45G | 91.37% | 93.98% | 49.80% |
| 16 | 49.06M | 7.36G | 48.16M | 6.80G | 92.43% | 93.08% | 49.58% |
| 17 | 49.68M | 7.45G | 48.76M | 6.83G | 91.70% | 92.97% | 48.04% |
| 18 | 51.72M | 7.76G | 50.86M | 7.22G | 93.12% | 94.82% | 48.22% |
| 19 | 49.36M | 7.40G | 48.33M | 6.77G | 91.47% | 94.00% | 49.50% |
| 20 | 49.65M | 7.45G | 48.75M | 6.85G | 91.95% | 93.92% | 50.00% |
| 21 | 48.83M | 7.32G | 48.02M | 6.79G | 92.70% | 95.11% | 48.22% |
| 22 | 48.69M | 7.30G | 47.79M | 6.71G | 91.82% | 94.23% | 50.24% |
| 23 | 47.71M | 7.16G | 46.93M | 6.62G | 92.45% | 93.39% | 49.32% |
| 24 | 47.23M | 7.08G | 46.42M | 6.57G | 92.80% | 95.04% | 48.87% |
| 25 | 47.87M | 7.18G | 47.09M | 6.65G | 92.65% | 94.91% | 48.65% |
| 26 | 47.49M | 7.12G | 46.62M | 6.60G | 92.60% | 94.89% | 48.77% |
| 27 | 47.31M | 7.10G | 46.47M | 6.55G | 92.34% | 95.01% | 48.89% |
| 28 | 46.09M | 6.91G | 45.28M | 6.35G | 91.80% | 94.29% | 49.58% |
| 29 | 46.14M | 6.92G | 45.42M | 6.47G | 93.46% | 95.07% | 48.48% |
| 30 | 50.48M | 7.57G | 49.64M | 7.04G | 93.02% | 94.79% | 48.68% |
| 31 | 45.30M | 6.80G | 44.54M | 6.36G | 93.53% | 95.01% | 48.44% |
| 32 | 49.86M | 7.48G | 49.05M | 6.98G | 93.39% | 94.96% | 48.01% |
| 33 | 48.99M | 7.35G | 48.00M | 6.75G | 91.88% | 93.68% | 50.53% |
| 34 | 48.60M | 7.29G | 47.67M | 6.66G | 91.40% | 94.07% | 47.60% |
| 35 | 46.27M | 6.94G | 45.46M | 6.41G | 92.40% | 94.15% | 48.89% |
| 36 | 49.52M | 7.43G | 48.59M | 6.75G | 90.91% | 93.19% | 50.26% |
| 37 | 49.89M | 7.48G | 48.95M | 6.83G | 91.32% | 93.86% | 49.79% |
| 38 | 47.41M | 7.11G | 46.49M | 6.54G | 91.94% | 93.85% | 49.72% |
| 39 | 50.21M | 7.53G | 49.17M | 6.89G | 91.51% | 93.76% | 49.85% |
| 40 | 48.80M | 7.32G | 47.87M | 6.71G | 91.61% | 94.84% | 48.71% |
| 41 | 48.47M | 7.27G | 47.70M | 6.76G | 93.00% | 95.18% | 49.35% |
| 42 | 46.79M | 7.02G | 46.01M | 6.53G | 93.05% | 95.01% | 49.09% |
| 43 | 43.28M | 6.49G | 42.35M | 6.06G | 93.35% | 94.82% | 48.35% |
| 44 | 47.58M | 7.14G | 46.55M | 6.47G | 90.61% | 93.79% | 46.81% |
| 45 | 44.56M | 6.68G | 43.77M | 6.16G | 92.23% | 94.07% | 49.86% |
| 46 | 48.47M | 7.27G | 47.69M | 6.73G | 92.57% | 95.09% | 49.08% |
| 47 | 44.75M | 6.71G | 44.03M | 6.26G | 93.26% | 95.14% | 48.63% |
| 48 | 48.21M | 7.23G | 47.14M | 6.59G | 91.19% | 93.84% | 47.90% |
| 49 | 47.94M | 7.19G | 46.91M | 6.58G | 91.44% | 93.84% | 45.86% |
| 50 | 46.35M | 6.95G | 45.64M | 6.53G | 93.89% | 95.15% | 49.19% |
| 51 | 47.26M | 7.09G | 46.31M | 6.55G | 92.42% | 93.19% | 49.98% |


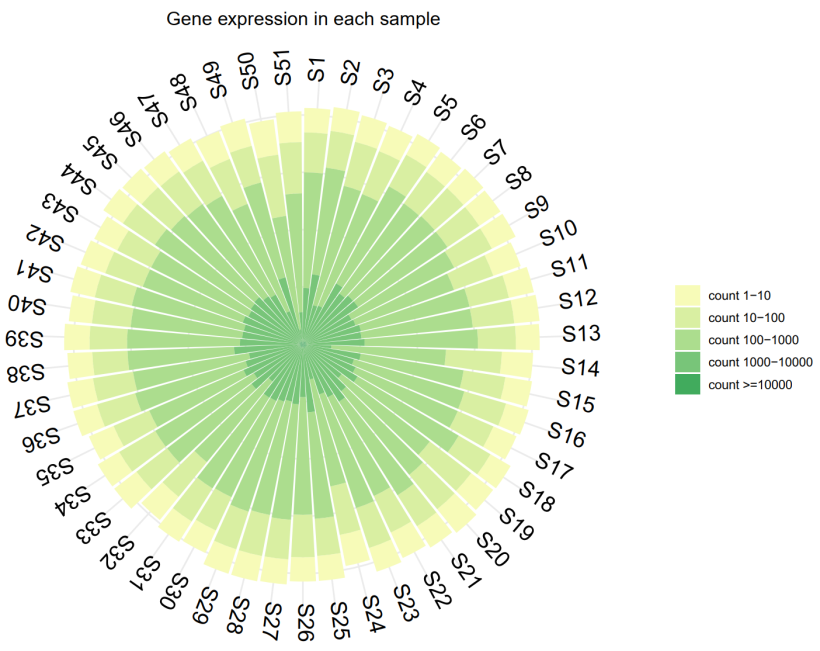


**Figure S3** Statistical diagram of the number of genes detected in each sample. Each fan on the graph represents the number of genes detected in a sample, with different colors indicating the range of gene numbers.


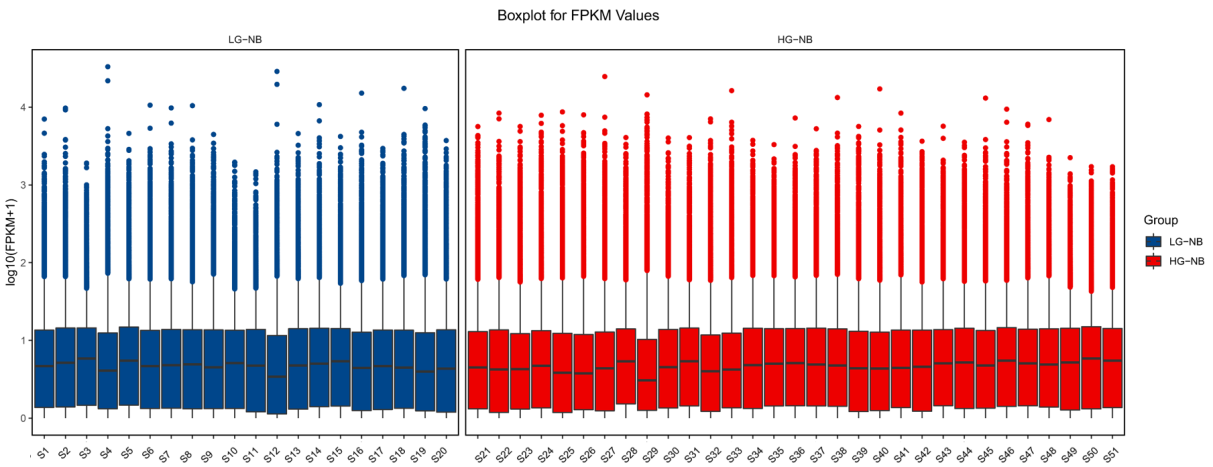


**Figure S4** Boxplots of FPKM values. The horizontal axis of the graph represents the sample names, while the vertical axis represents log_10_(FPKM+1). Each box plot in the region corresponds to five statistics (from top to bottom: maximum value, third quartile, median, first quartile, and minimum value).


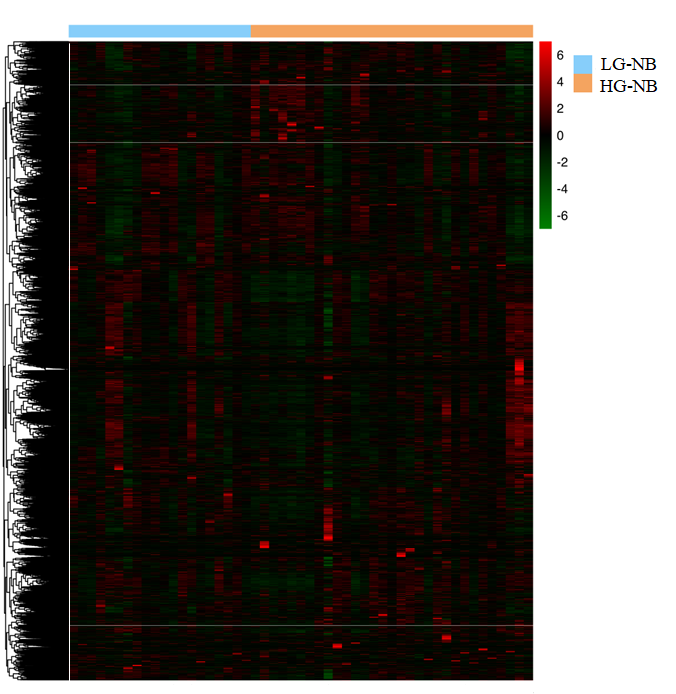


**Figure S5** Heatmap of all genes between HG-NB and LG-NB in transcriptomics.

| Id | Term |
| --- | --- |
| hsa04024 | cAMP signaling pathway |
| hsa04061 | Viral protein interaction with cytokine and cytokine receptor |
| hsa05144 | Malaria |
| hsa05143 | African trypanosomiasis |
| hsa04923 | Regulation of lipolysis in adipocytes |
| hsa05033 | Nicotine addiction |
| hsa04060 | Cytokine-cytokine receptor interaction |
| hsa04080 | Neuroactive ligand-receptor interaction |
| hsa04927 | Cortisol synthesis and secretion |
| hsa05414 | Dilated cardiomyopathy |
| hsa03320 | PPAR signaling pathway |
| hsa05323 | Rheumatoid arthritis |
| hsa04512 | ECM-receptor interaction |
| hsa04657 | IL-17 signaling pathway |
| hsa05146 | Amoebiasis |
| hsa05322 | Systemic lupus erythematosus |
| hsa05410 | Hypertrophic cardiomyopathy |
| hsa04380 | Osteoclast differentiation |
| hsa04062 | Chemokine signaling pathway |
| hsa05202 | Transcriptional misregulation in cancer |

**Table S5** Annotated table of each pathway in KEGG map.

**Table S6** **Primers used by RT-PCR to verify the significant genes**

| **Gene** | **Forward** | **Reverse** |
| --- | --- | --- |
| *MGST1* | TATGCCATCGTTTGGCACAG | TCCAAAATGAAAGAAAGTTTCCGTG |
| *SERPINE1* | AATGCCCTCTACTTCAACGG | AGGGGCTCTTGGACTTACTATAG |
| *IGF2* | GCTTCCCCCTAACACACCAAG | GGGGACCAATTTGTGAGGAGG |
| *CIP2A* | GGTGAGCATGAATAAGGGGAC | TGGCTCATCCCAGATGGTTG |
| *CHL1* | GTTTGGTACATTTAGGGTGGG | AAGAAACCCTGGTCAAAGC |
| *ERBB3* | TCAAAGGTGCCTGACTCTTCC | CCTGGGGGTTGAGAAGAAAGG |


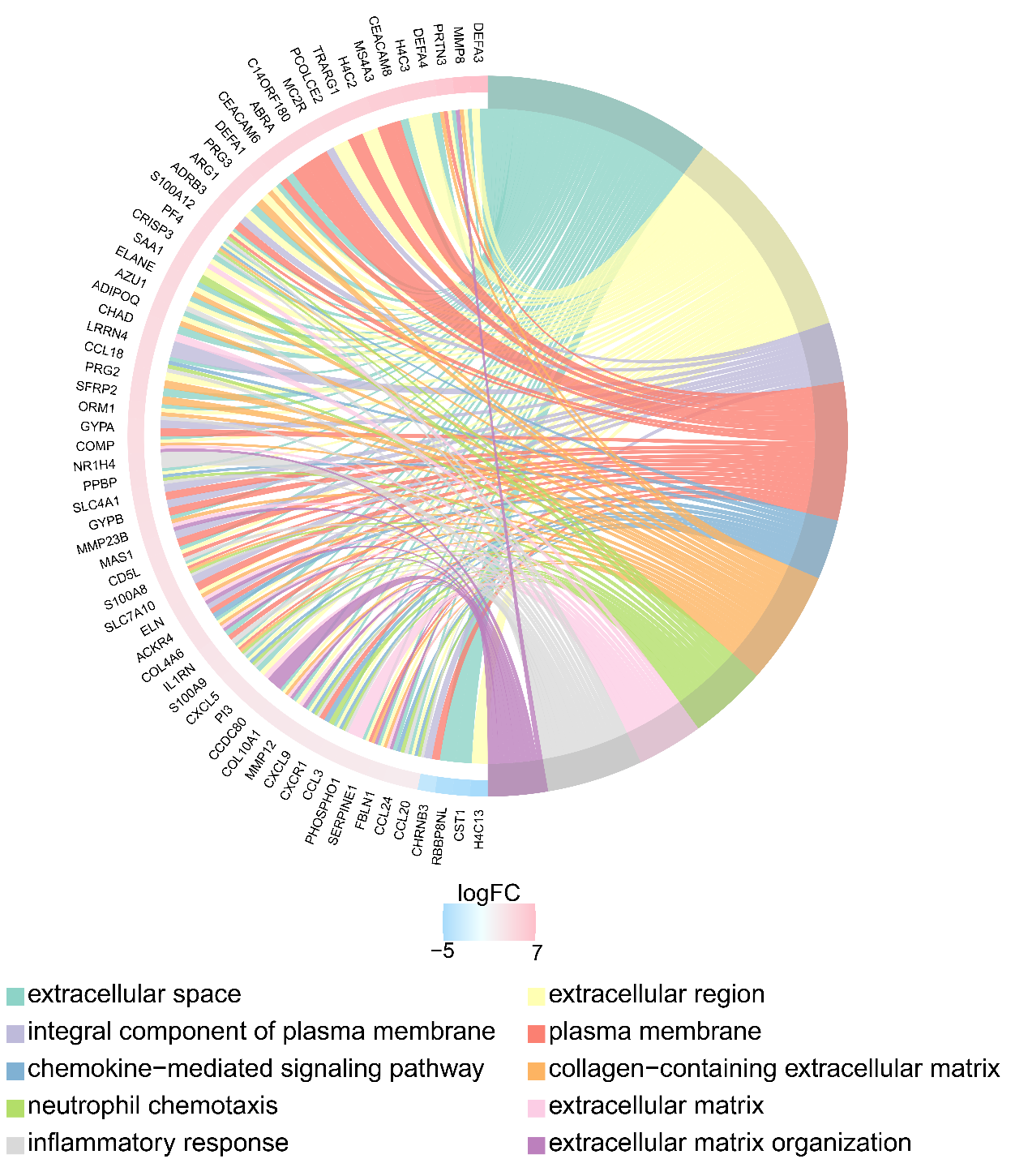


**Figure S6** GO chord diagram between HG-NB and LG-NB in transcriptomics.


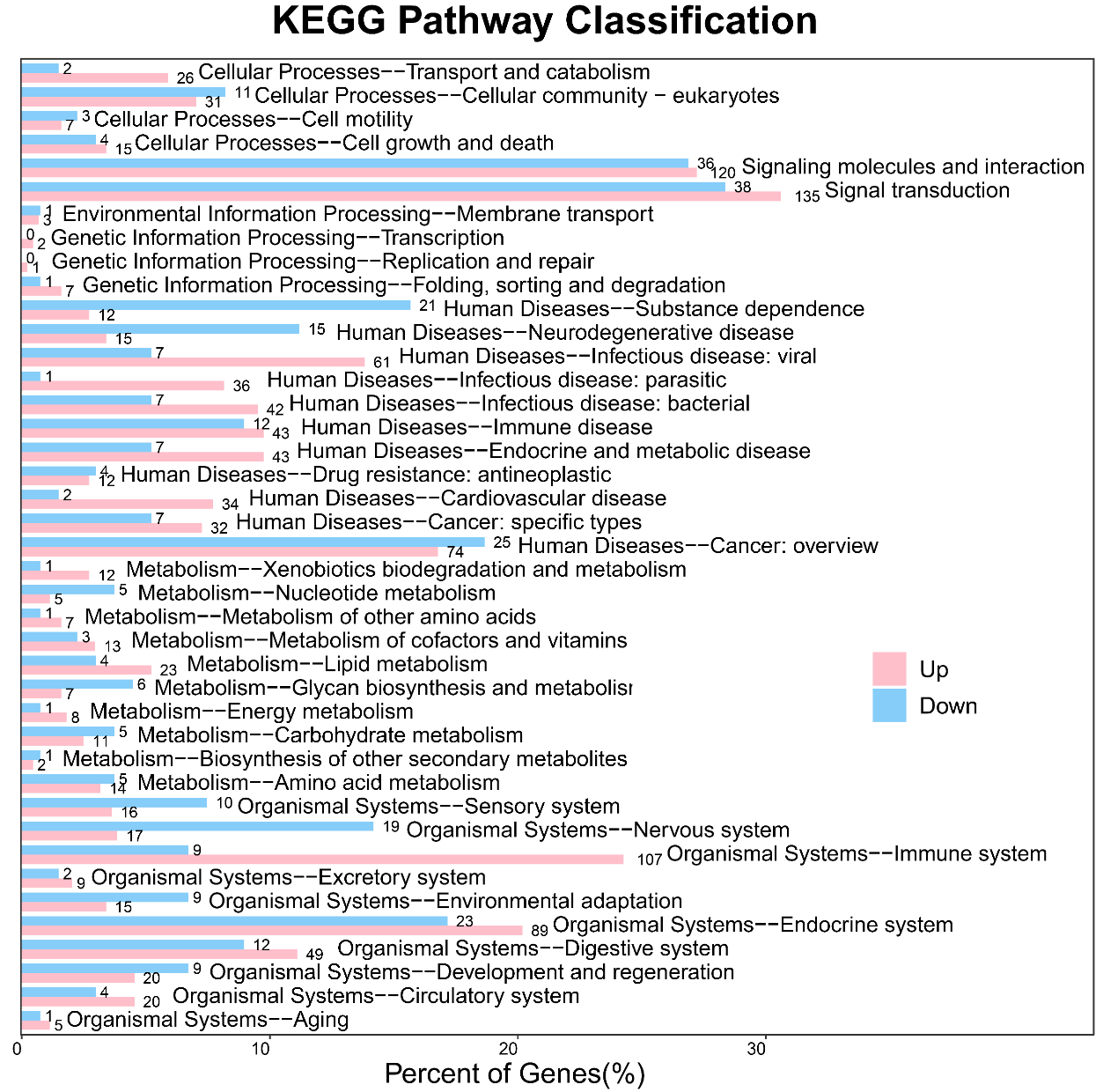


**Figure S7** KEGG classification analysis between HG-NB and LG-NB in transcriptomics.


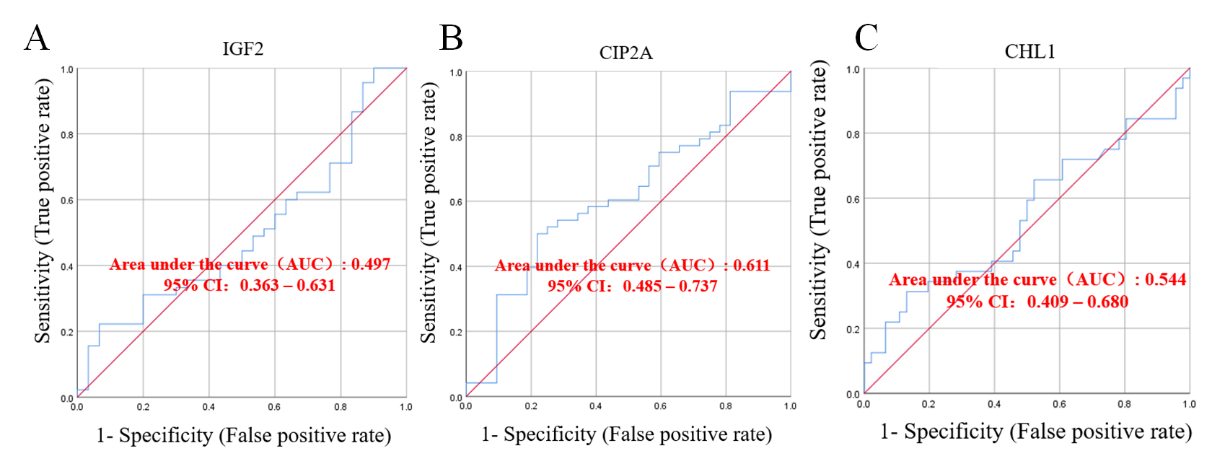


**Figure S8** The ROC curve of three biomarkers *IGF2* (A), *CIP2A* (B), *CHL1* (C).
